# Augmented transcripts of kidney injury markers and renin angiotensin system in urine samples of overweight young adults

**DOI:** 10.1038/s41598-020-78382-3

**Published:** 2020-12-03

**Authors:** Patricia Rivera, Catalina Miranda, Nicole Roldán, Aaron Guerrero, Javier Olave, Pilar Cárdenas, Quynh My Nguyen, Modar Kassan, Alexis A. Gonzalez

**Affiliations:** 1 Institute of Chemistry, Pontificia Universidad Católica de Valparaíso, Valparaíso, Chile; 2 Skaggs School of Pharmacy and Pharmaceutical Sciences, University of California, San Diego, San Diego, CA USA; 3 Cardiovascular Division, Department of Medicine, Abboud Cardiovascular Research Center, University of Iowa Carver College of Medicine, Iowa City, IA USA

**Keywords:** Biochemistry, Physiology, Biomarkers, Molecular medicine, Risk factors

## Abstract

Obesity has been firmly established as a major risk factor for common disease states including hypertension, type 2 diabetes mellitus, and chronic kidney disease. Increased body mass index (BMI) contributes to the activation of both the systemic and intra-tubular renin angiotensin systems (RAS), which are in turn associated with increased blood pressure (BP) and kidney damage. In this cross-sectional study, 43 subjects of normal or increased body weight were examined in order to determine the correlation of BMI or body fat mass (BFM) with blood pressure, fasting blood glucose (FBG), and urinary kidney injury markers such as interleukin-18 (IL-18), connective tissue growth factor (CTGF), neutrophil gelatinase-associated lipocalin, and kidney injury molecule-1 (KIM-1). Our results showed that: (1) subjects with increased body weight showed significantly higher BP, BFM, total body water and metabolic age; (2) BMI was positively correlated to both systolic (R^2^ = 0.1384, *P* = 0.01) and diastolic BP (R^2^ = 0.2437, *P* = 0.0008); (3) BFM was positively correlated to DBP (R^2^ = 0.1232, *P* = 0.02) and partially correlated to urine protein (R^2^ = 0.047, *P* = 0.12) and FBG (R^2^ = 0.07, *P* = 0.06); (4) overweight young adults had higher urinary mRNA levels of renin, angiotensinogen, IL-18 and CTGF. These suggest that BMI directly affects BP, kidney injury markers, and the activation of the intra-tubular RAS even in normotensive young adults. Given that BMI measurements and urine analyses are non-invasive, our findings may pave the way to developing a new and simple method of screening for the risk of chronic kidney disease in adults.

## Introduction

Obesity is an epidemic affecting more than 1.4 billion adults worldwide^1^ and 17% of youths in the United States^2^. In individuals classified as overweight (BMI = 25–29.9 kg/m^2^), there is a higher probability of acquiring metabolic syndrome, which contributes to the development of Type 2 diabetes, hypertension and cardiovascular disease^3^. Type 2 diabetes and hypertension are the main risk factors for CKD^4,5^. Obesity is characterized by activation of the systemic renin angiotensin system (RAS), a key regulator of blood pressure, fluid and electrolyte balance^6,7^. Under pathological conditions, RAS activation plays an important role in the progression of hypertension. The systemic RAS is controlled by renin production in the juxtaglomerular (JG) cells of the kidney, which release renin into the circulation. Once in the systemic circulation, renin acts on angiotensinogen (AGT) produced by the liver to form angiotensin (Ang) I. Ang I is cleaved and converted to Ang II by angiotensin converting enzyme (ACE) in the lungs. Finally, Ang II promotes vasoconstriction and aldosterone release by the adrenals, thus promoting sodium reabsorption^8^.

It has been reported that systemic RAS activity correlates positively with body weight and body fat^7,9,10^. Adipocytes have been suggested to possess an additional RAS and to generate angiotensin peptides which serve as a source for systemic RAS components^11^. Because of this, increases in adipose tissue, as seen in overweight and obese individuals, may lead to an increase in blood pressure.

The RAS components are also expressed in the kidneys and are able to form Ang II^12,13^. Renin and ACE are expressed in collecting ducts^14,15^ while AGT is expressed in proximal tubules^16^. The local availability of AGT allows renin to form Ang I, which is converted to Ang II by ACE. In contrast to the inhibitory effect that Ang II exerts on JG renin, Ang II stimulates the expression of renin in the collecting duct and of AGT in proximal tubules, leading to further intratubular Ang II formation^17–20^. Substantial evidence supports the critical role that activation of this intra-tubular RAS has in the development and maintenance of hypertension^21–23^ and CKD^11,24^. Urinary renin and AGT have been proposed as biomarkers for intra-renal status of RAS activation^25,26^. Furthermore, the presence of urinary AGT precedes the development of Stage 3 CKD in patients with type 1 diabetes^27^.

Tubular damage markers have been used to assess several forms of kidney diseases^28–31^. Candidate biomarkers of early kidney injury have been proposed to be segment-specific in the nephron^32^. Among them, neutrophil gelatinase-associated lipocalcin (NGAL) and kidney injury molecule-1 (KIM-1) have emerged as promising biomarkers for early renal injury of the proximal and distal tubules. Interleukin 18 (IL-18) has been shown to be an early biomarker of tubular injury^32^. Connecting tissue growth factor (CTGF) is also associated with early profibrotic signals that will eventually lead to tubule-interstitial fibrosis^33^.

In this study we measured the urinary mRNA levels of renin, AGT and kidney injury markers in young adults who are overweight or of normal weight, to determine if these measurements accurately reflect intra-tubular RAS status. Our non-invasive approach included anthropometry, urine collection, bioelectrical impedance analysis, blood pressure, fasting blood glucose levels (FBG), and additional biometric parameters. We found not only that urinary mRNA for renin and AGT were higher in subjects within the overweight range or above (BMI ≥ 25), but IL-18 and CTGF mRNA levels were augmented as well. Interestingly, systolic and diastolic blood pressure (BP) correlated positively with BMI, although none of the individuals were diagnosed with clinical hypertension.

## Materials and methods

### Description of the study population

**Figure 1:**
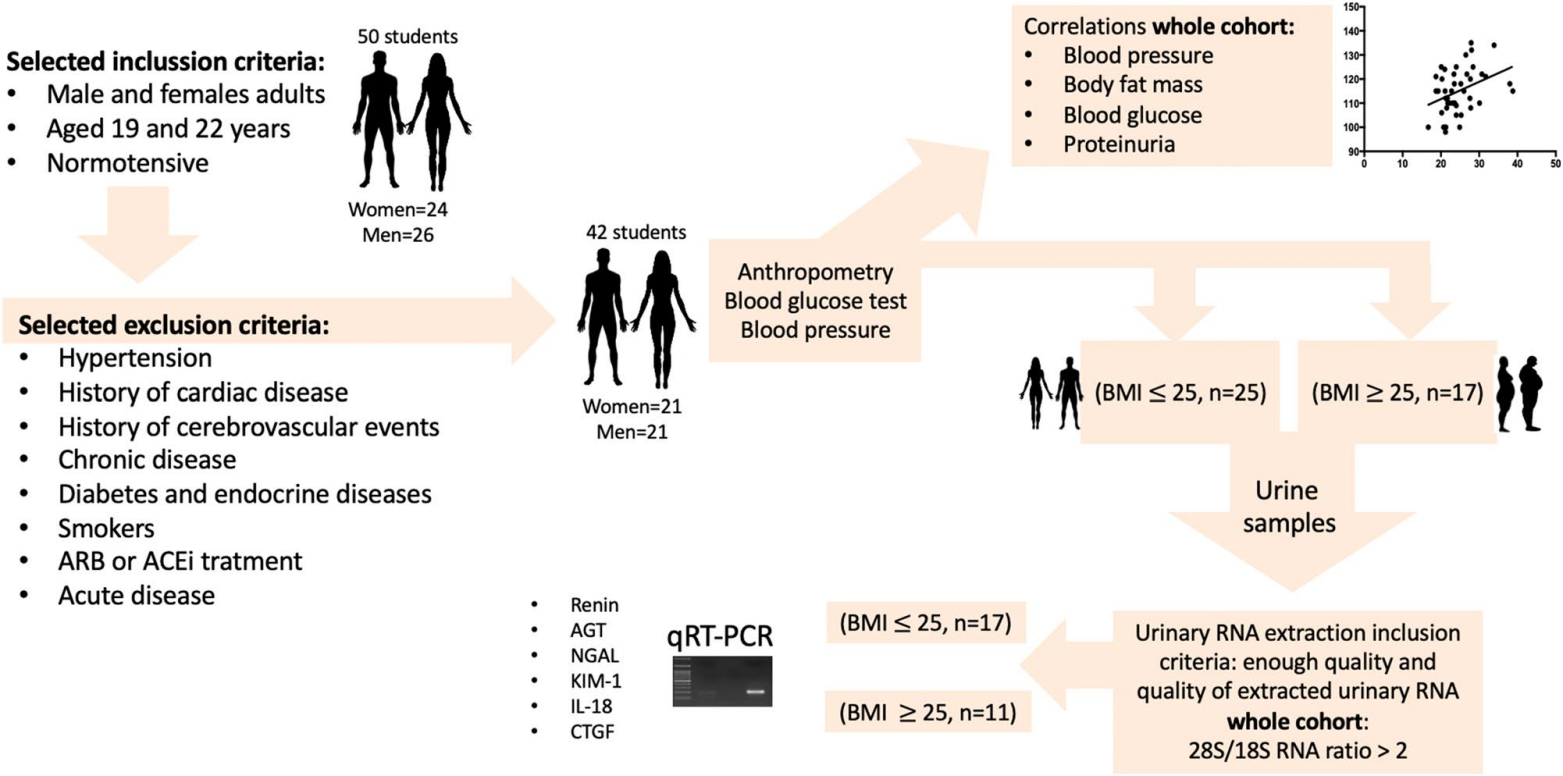
Schematic workflow of inclusion and exclusion criteria for the study. A cohort of 50 students were initially selected. Implementation of exclusion criteria left 42 students, upon whom we performed anthropometric analysis, fasting blood glucose measurements, urine biochemical analysis and blood pressure determinations. After inclusion and exclusion criteria for RNA quality, 28 students were categorized as normal weight or overweight (BMI ≥ 25). After total RNA extractions, the expression levels of mRNA transcripts of renin, angiotensinogen (AGT), neutrophil gelatinase-associated lipocalcin (NGAL), kidney injury molecule-1 (KIM-1), interleukin 18 (IL-18) and connecting tissue growth factor (CTGF) were analyzed.

Initially, 50 students aged 19–22 years old were recruited, and their written informed consent was obtained. Eight patients were subsequently excluded due to associated diseases (Fig. 1), leaving 42 individuals in the remainder of the study. These patients met the following inclusion criteria: non-smoking, normotensive, afebrile, no acute or chronic infection, and no treatment with ARBs, ACE inhibitors or anti-diabetic drugs in the preceding 4 weeks. Patients with a history of cardiac or cerebrovascular events and endocrine diseases were also excluded. These exclusion/inclusion criteria are shown in Fig. 1. Clinical characteristics are summarized in Table 1.

**Table 1:** Clinical parameters of individuals in normal weight and overweight groups.

Clinical characteristic	Normal weight n = 25	Overweight n = 17
Age	21.2 ± 1.2	20.7 ± 0.7
Women (n)	12	9
Men (n)	13	8
Systolic blood pressure (mmHg)	111.7 ± 1.6	120 ± 2.1*
Diastolic blood pressure (mmHg)	68.1 ± 1.4	78.5 ± 2.5*
Fasting blood glucose (mg/dL)	91.5 ± 1.2	92.1 ± 1.6
Blood O_2_ saturation (%)	98.2 ± 0.2	97.8 ± 0.2
Heart rate (beats / min)	75.3 ± 1.5	77.3 ± 2.9
Height (cm)	165.5 ± 1.4	166.4 ± 1.5
Weight (kg)	59.1 ± 1.3	81.3 ± 2.6**
BMI (kg/m^2^)	21.4 ± 1.3	29.4 ± 0.9***
Basal metabolic rate (Kcal/kg)	1,461 ± 37	1,696 ± 49
Visceral fat (%)	19 ± 2	32 ± 2*
Total water (kg)	34 ± 1	40 ± 1*
Visceral fat index	1.78 ± 0.32	6.06 ± 0.57***
Metabolic age (years)	17 ± 1	34 ± 2***
Urine pH	5.84 ± 0.11	5.94 ± 0.12

*P < 0.05; **P < 0.01; ***P < 0.001.

The ethics committee which approved the study was the *bioethical committee of the Pontificia Universidad Católica de Valparaiso, Chile*. All experiments were performed in accordance with relevant guidelines and regulations of the *bioethical committee of the Pontificia Universidad Católica de Valparaiso, Chile*. Individuals were classified as overweight according to body mass index (BMI) ≥ 25 or waist ≥ 85 cm (women) or ≥ 90 cm (men).

### Anthropometric measurements

Measurements of body weight, BMI, and height were performed on the day of biochemical analysis. Body composition was assessed by bio-impedance analysis (Tanita BC 420 S MA Class III), which calculated percentage of fat and index of fat mass and water content among other parameters (Table 1).

### Blood pressure measurements

Measurements were performed by the auscultatory method using a certified mercury sphygmomanometer in a quiet room with comfortable temperature. The subject was asked not to talk during measurements. The arm was bare and placed at heart level. A standard cuff (12.5 cm bladder) was used and fitted appropriately to the subject’s arm size. Systolic and diastolic blood pressure was recorded as the mean value of 3 measurements. Because obese individuals may be wrongly identified as hypertensive due to ill-fitting blood pressure cuffs that are too small, we verified that all six of the obese subjects had arm circumferences of less than 33 cm (28.7 ± 3 cm), which is compatible with the standard cuff^34^.

### Urine analysis

Urine samples (20–30 mL) were collected in the morning. The urine was deposited in a sterile container which was kept at 4 °C in the laboratory for subsequent RNA extraction the same day. An aliquot of the sample (1 mL) was observed microscopically to evaluate for the presence of bacteria. Samples were discarded if they contained bacteria. Chemical parameters were measured in each sample using test strips (Combina 13, Biotek solutions, Macedonia), and included glucose, ketones, pH, proteins, nitrites, leukocytes, relative density, urobilinogen, bilirubin, and the presence of blood (i.e. hemoglobin or erythrocytes). Urinary protein concentration was measured after a 10 min sample centrifugation.

### RNA extractions

Urine samples (20–50 mL) were centrifuged at 3000 g for 10 min at 4 °C. Samples were maintained on ice (4 °C) at all times to avoid RNA degradation. The supernatant was removed, and the pellet was re-suspended in 750 µL of Trizol solution (Invitrogen, Carlsbad, CA) to obtain a pink cloudy solution. Total RNA extraction was performed using the Trizol-chloroform method according to the manufacturer's instructions. RNA quantification was performed by absorption spectrophotometry using the Epoch Spectrophotometer System (BioTek, UK) measuring at 260 and 280 nm.

### RNA integrity

The 28S/18S ribosomal RNA (rRNA) ratio (> 2) was used to assess the quality of total RNA purified from each sample. Total RNA integrity was also evaluated in a 0.8% agarose gel prepared in 1X TAE buffer with nuclease free and deionized water. RNA (500 ng per well) was loaded at a final volume of 10 µL. Ladder 1 kb (PCR 100 bp Low Ladder P1473, Sigma) plus (3 µL) was loaded and the samples were run at 80 V for 45 min. The criteria used to establish RNA integrity was the presence of 28S (~ 5070 kb) and 18S (1869 kb) rRNA bands at an 28S/18S ratio of ~ 2.7. The intensity of each band was analyzed using Image J. In addition, we determine 18S rRNA levels to ensure polymerase chain reaction (PCR) quality and reproducibility considering the effect of total RNA degradation.

### Primers design and quantitative PCR (qPCR) standardization

RNA sequences for human renin, AGT, IL-18, CTGF, NGAL and KIM-1 were BLASTed using NCBI software. After the analysis of sequence complementarity using Primer-BLAST software, we proceeded to design the specific primers in the Primer3 website. Primers are shown in Table 2. Total RNA samples (0.35 µg) were used as templates for the synthesis of the first strand DNA using the abm 5× all in one RT MAstermix kit (Abm, Canada) and M-MLV reverse transcriptase. Complementary DNA (cDNA) samples were stored at − 20 °C. Quantitative PCR was assessed with the Fast Start DNA Sybr Green kit (SIGMA, San Louis, Missouri). The abundance of mRNA for each transcript was assessed by Ct values using the formula 2^−ΔΔCT^ and the ratio of the gene of interest versus 18S rRNA abundance.

**Table 2:** Primers used for amplification of mRNA using qPCR.

	Sequence	Length (bp)	PCR product (bp)	Tm (°C)	GC%
**AGT**					
FW	TATGATCAAAGCGCCACTGC	20	191	58.9	50
RV	AGAAAAGGTGGGAGACTGGG	20		58.9	50
**Renin**					
FW	GTTTGGAGAGGTCACGGAGA	20	196	59.1	55
RV	CAGCGATTGGGAATTCTCGG	20		59.1	55
**IL-18**					
FW	AAGACCAGCCTGACCACAT	20	197	59.2	50
RV	ACCCGTTGTTGTCGTTTTGA	20		59.1	50
**CTGF**					
FW	TACCAATGACAACGCCTCCT	20	207	59.2	50
RV	CCGTCGGTACATACTCCACA	20		58.2	50
**NGAL**					
FW	TTCCTCGGCCCTGAAATCAT	20	179	56.5	50
RV	ACCACATACCACTTCCCCTG	20		56.7	55
**KIM-1**					
FW	TGGAACCCACGTCACCTATC	20	205	56.5	55
RV	TGGAGTAGTCGTGACCTTGG	20		56.1	55
**AQP-2**					
FW	ACC TGGCTGTCAATGCTCTC	24	190	62.2	50
RV	GCCGGTGTAATGGATCCCAA	24		61.7	50
**18S**					
FW	AAACGGCTACCACATCCAAGGAAG	24	159	63.2	50
RV	GCCCTCCAATGGATCCTCGTTAAA	24		62.7	50

### Statistical analysis

Statistical analyses were performed using GraphPad Prism Software Version 6 (GraphPad Software, Inc., La Jolla, CA, USA). Clinical characteristics and laboratory data of all patients at baseline are reported as the mean ± S.E.M. Comparisons between normal weight (n = 25) and overweight (n = 17) groups were performed using non-paired (one-tailed) t-test. Normal distribution of each parameter analyzed was tested by using Shapiro–Wilk. The correlation coefficient was used to verify the relationship between systolic or diastolic blood pressure and BMI, or fasting blood glucose and proteinuria in the population analyzed. A *P* value < 0.05 was considered statistically significant.

## Results

### Significant association of body mass index and blood pressure among the study population

**Figure 2:**
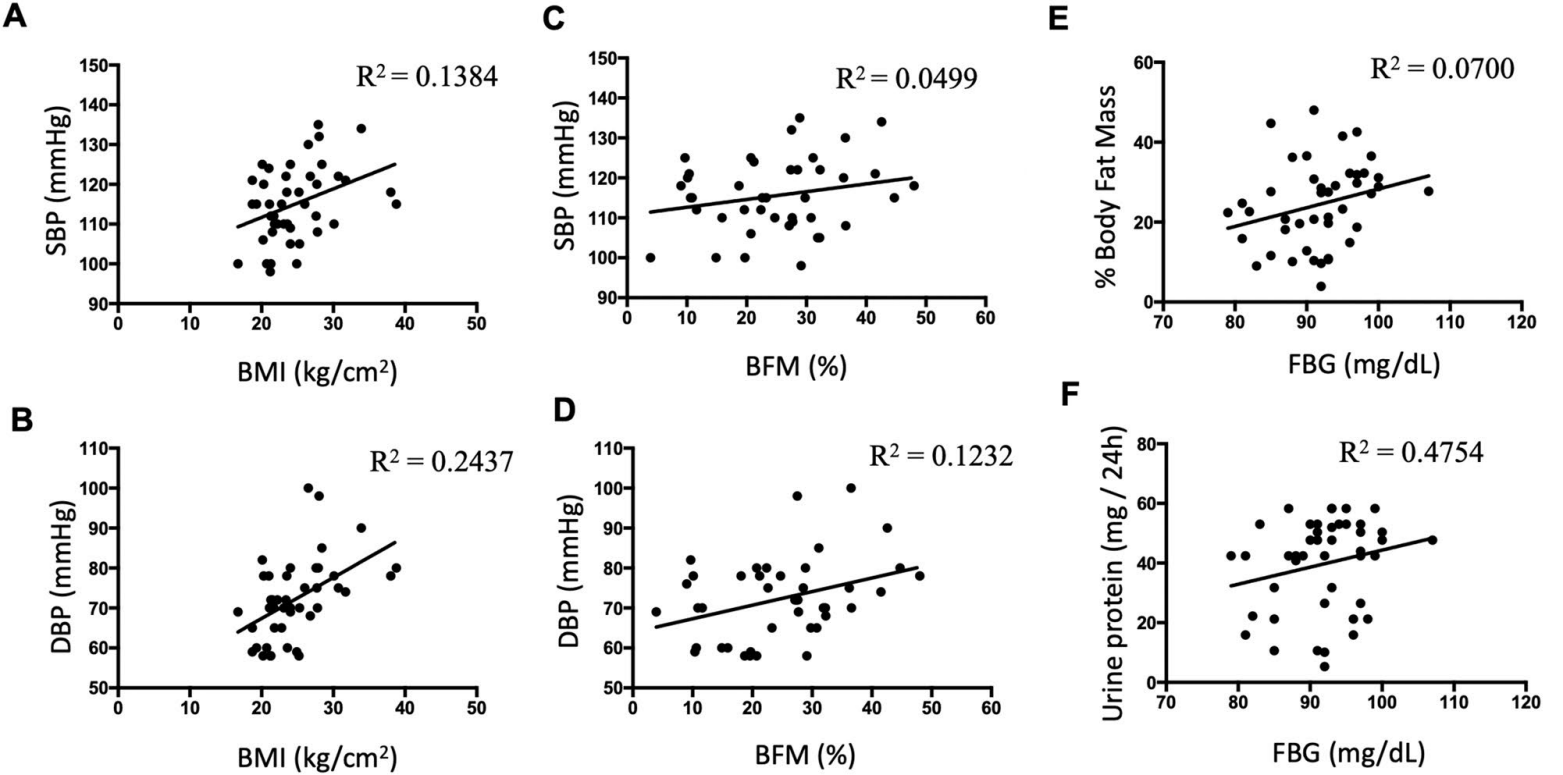
Linear regression analysis of blood pressure versus body mass index (BMI), percentage of body fat mass (BFM%) versus fasting blood glucose (FBG) and 24 h urinary protein versus FBG in the study population. (**A**) Significant deviation from zero was observed when analyzing systolic BP versus BMI. (**B**) Similarly, significant deviation from zero was observed when analyzing diastolic BP versus BMI. (**C**) Linear regression of percentage of body fat mass (BFM%) and systolic BP showed no significant correlation. (**D**) Diastolic blood pressure showed a significant correlation with BFM%. (**E**) Linear regression of BFM% and fasting blood glucose showed no significant deviation from zero; however, it did show a positive slope. (**F**) A weak but positive correlation between fasting blood glucose and proteinuria was also observed, n = 42.

Linear regression of BP and BMI in the overall sample showed significant deviation from zero when analyzing systolic BP versus BMI (95% CI slope: 0.15–1.28; best-fit values ± SE: slope: 0.7177 ± 0.2797, R^2^ = 0.1384, *P* = 0.014) and diastolic BP versus BMI (95% CI slope: 0.45–1.59; best-fit values ± SE: slope: 1.02 ± 0.28, R^2^ = 0.2437, *P* = 0.0008). The association of BMI and SBP is shown in Fig. 2A,B. Linear regression of percentage of body fat mass (BFM%) and systolic BP showed no significant correlations (95% CI slope: − 0.08 to 0.47; best-fit values ± SE: slope: 0.1952 ± 0.1363, R^2^ = 0.0499, *P* = 0.1602); however, diastolic blood pressure showed a significant correlation (95% CI slope: 0.05–0.63; best-fit values ± SE: slope: 0.3403 ± 0.1435, R^2^ = 0.1232, *P* = 0.0227), see Fig. 2C,D.

### Association of fasting blood glucose with body fat mass and urinary protein among the study population

BMI and adiposity show a high degree of correlation in children and adolescents^35,36^. Linear regression of BFM% and BMI correlations in the overall sample showed significant deviation from zero (95% CI slope: 1.32–2.13; best-fit values ± SE: slope: 1.72 ± 0.21, R^2^ = 0.62, *P* = 0.000012). We examined whether there was an association between BFM% and fasting blood glucose in the study population. Although linear regression of BFM% and fasting blood glucose correlations in the overall sample showed no significant deviation from zero (95% CI slope: − 0.07 to 1.01; best-fit values ± SE: slope: 0.46 ± 0.26), it did show a positive slope Fig. 2E with R^2^ = 0.07 (*P* = 0.06). Similarly, there was a weak but positive correlation between fasting blood glucose and proteinuria (95% CI slope: − 0.22 to 1.36; best-fit values ± SE: slope: 0.57 ± 0.39, *P* = 0.11, R^2^ = 0.4754, Fig. 2F). Although six subjects were obese, we did not perform a separate analysis for this group because all patients with obesity were collectively included in the overweight group (BMI ≥ 25).

### Biochemical characterization of urine samples in the study population

**Figure 3:**
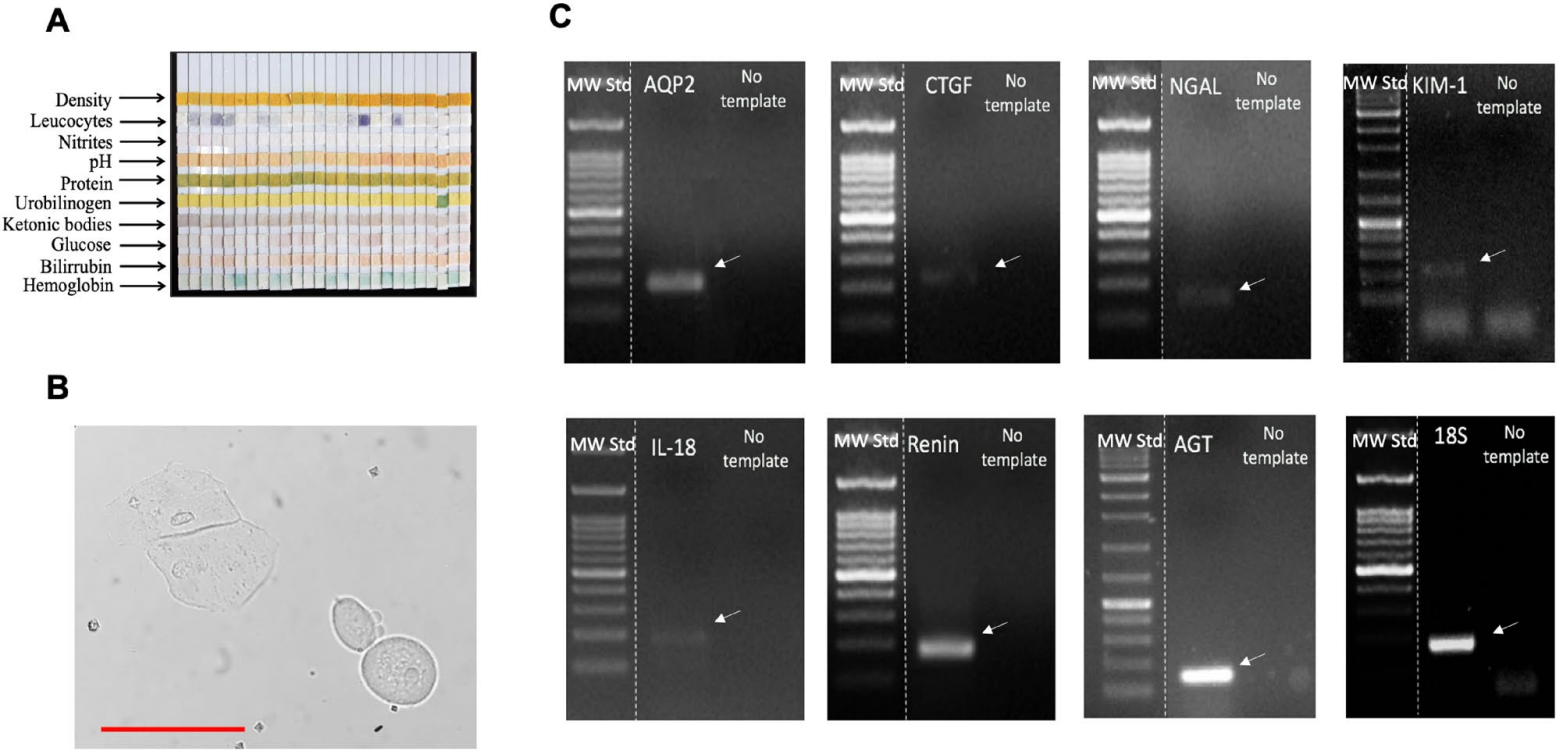
(**A**) Representative urine test strips showing indicators of density, leucocytes, nitrites, pH, protein levels, urobilinogen, ketonic bodies, glucose, bilirubin and hemoglobin. (**B**) Representative image of desquamated cells present in urine from a healthy subject. Scale bar 10 microns. (**C**) Images of electrophoretic gels showing PCR products of each gene analyzed. Arrows indicate the specific PCR product band. First lane in each gel represent the molecular weight standard that ranged from 100 to 1200 bp. The intense band represents 500 base pairs. Renin and AGT band intensities were subjectively as high as aquaporin-2 (AQP-2), a constitutive gene expressed in the collecting duct. Injury markers were less abundant as judged by band intensity.

Urine analyses were performed in the population. A representative set of urine strips are shown in Fig. 3A. No differences were found in urine specific density (normal weight: 1.03 ± 0.02 versus overweight: 1.01 ± 0.10). Leukocytes (25–75 leukocytes/µL) were detected in 3 individuals with normal weight (11%) and 3 overweight individuals (17%). No detectable levels of nitrites were found by this method. Values of urine pH are shown in Table 1. Urobilinogen was detected in 5 individuals with normal weight (15%) and 6 overweight subjects (35%). Hemoglobin was present in 34% of the population with normal weight versus 52% of overweight individuals. The presence of epithelial cells was assessed by analyzing urine samples from all individuals in the study. Figure 3B provides a representative image of desquamative cells from urinary tract epithelia. We did not observe a difference in the number of desquamative cells between the groups. Renin and AGT band intensities were subjectively as high as aquaporin-2 (AQP-2), a constitutive gene expressed in the collecting duct. Injury markers were less abundant as judged by band intensity (Fig. 3C).

### Increased urinary mRNA levels of renin and angiotensinogen in overweight young adults

**Figure 4:**
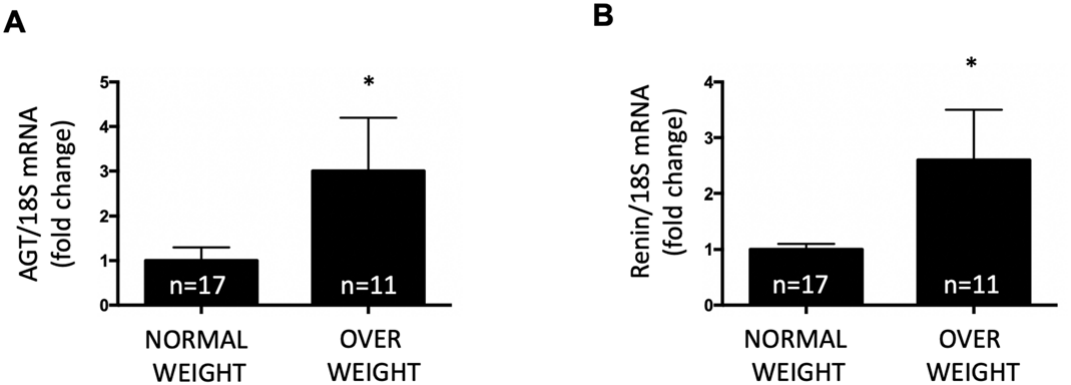
Expression levels of mRNA transcripts of angiotensinogen (AGT) and renin in individuals having normal weight and in overweight subjects. The criteria to conduct the mRNA analysis using qPCR was the presence of 28S (~ 5070 kb) and 18S (1869 kb) rRNA bands in an electrophoretic gel of integrity giving a 28S/18S ratio of ~ 2.7. After these exclusion criteria, only 28 urine samples were analyzed. As shown in the figure, overweight individuals showed higher expression levels of AGT (**A**) and renin (**B**) as compared to subjects with normal weight. *P < 0.05.

To determine the status of the intra-tubular RAS, we evaluated the mRNA levels of renin and AGT in the study groups. As previously described, the criteria used to establish RNA integrity was the presence of the 28S and 18S rRNA bands with a ratio ~ 2.0. Expression levels are presented as biomarker versus 18S mRNA. Therefore, the mRNA of interest is normalized with a housekeeping gene (18S RNA) independently of the number of desquamated cells obtained. After two urinary sample collections and implementation of the inclusion/exclusion criteria for RNA integrity, 17 individuals with normal weight and 11 of the overweight group were selected. As shown in Fig. 4, AGT mRNA levels were significantly higher in overweight individuals than in normal weight individuals (3.0 ± 1.2 vs. 1.0 ± 0.3, *P* < 0.05). Renin mRNA levels were also greater in this group (2.6 ± 0.9 vs. 1.0 ± 0.2, *P* < 0.05).

### Overweight young adults showed higher urinary mRNA levels of injury markers IL-18 and CTGF

**Figure 5:**
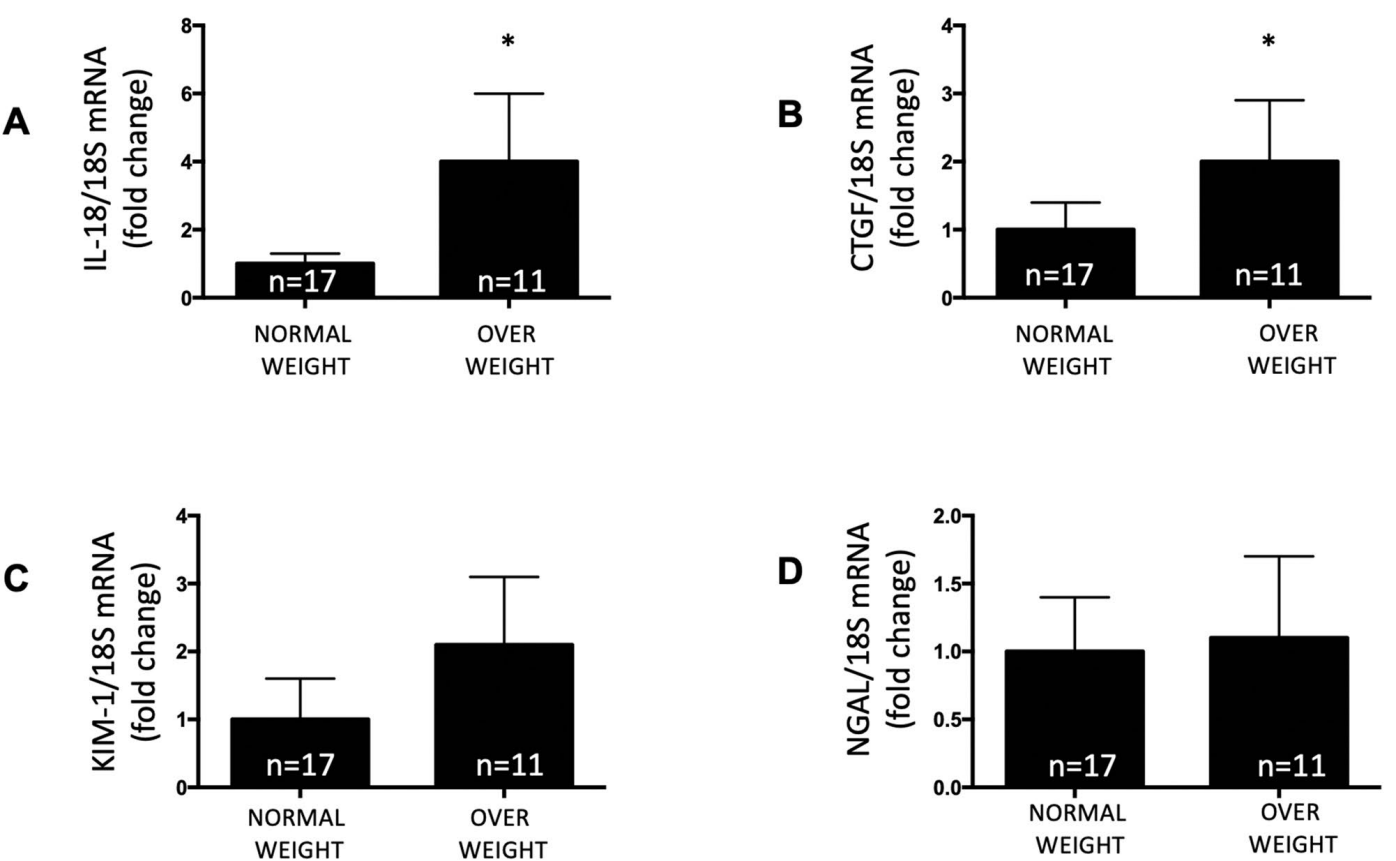
Expression levels for mRNA transcripts of kidney injury markers. (**A**) Interleukin 18 (IL-18), (**B**) Connecting tissue growth factor (CTGF), (**C**) Kidney injury marker (KIM-1) and (**D**) Gelatinase-associated lipocalcin (NGAL) in normal and overweight individuals. Higher expression levels of IL-80 and CTGF were seen in the overweight group (*P* < 0.05). No differences were found in renin as compared to subjects with normal weight. *P < 0.05.

Obesity is related to Type 2 diabetes and hypertension, both of which are associated with kidney damage. We examined urinary mRNA levels of IL-18, CTGF, NGAL and KIM-1, all of which are candidate biomarkers for tubular damage. Figure 4 shows the mRNA levels of each biomarker versus 18S mRNA. IL-18 and CTGF levels were significantly higher in overweight individuals (4.1 ± 2.1 vs. 1.0 ± 0.3, *P* < 0.05 and 2.0 ± 0.9 vs. 1.0 ± 0.4, *P* < 0.05, respectively) (Fig. 5A,B). We did not find a significant difference in the expression of KIM-1 and NGAL between the two groups (Fig. 5C,D).

## Discussion

Obesity is linked to hypertension and Type 2 diabetes, the most important causes of CKD^4,37^. In obesity, systemic RAS activity is increased, promoting sodium retention and elevated blood pressure^7,10^. RAS is also activated in visceral fat. Sustained stimulation of systemic RAS leads to the activation of intra-renal and intra-tubular RAS^38^. Ang II, the final effector of systemic RAS, promotes the upregulation of all components of intra-tubular RAS including AGT, ACE and renin. Augmented synthesis and secretion of AGT produced locally by proximal tubule cells serves as a substrate for renin synthetized by the collecting duct. This provides a pathway for the local generation of angiotensin I, such that subsequent conversion to Ang II by ACE is abundant in the kidney^11^. Additional intra-tubular Ang II formation stimulates sodium retention and synthesis of profibrotic factors such as CTGF and IL-18 (among others), promoting proliferation, fibrosis, and renal injury^33,39,40^.

In this study, we demonstrate that overweight subjects had higher levels of transcripts of urinary RAS components, renin and AGT (Fig. 4A,B), in addition to greater levels of urinary markers for tubular damage, IL-18 and CTGF (Fig. 5A,B). Although IL-18 has been described as marker for kidney injury^33,39,40^, it is possible that higher urinary IL-18 mRNA levels reflect filtered mRNA from a systemic inflammatory response in obesity. Although the same can be expected of CTGF, most of the evidence suggests that elevated urinary CTGF is more likely to be caused by local production and tubular dysfunction in kidney disease^41,42^. We further investigate the expression levels of more specific kidney injury markers, KIM-1 and NGAL. KIM-1 is a transmembrane glycoprotein. Protein expression is not detectable in the normal kidney; however, it can be detected in the urine samples of patients with acute kidney injury (AKI) and has been proposed to be a biomarker for tubular injury^43^. As shown in Fig. 3C, low expression levels of KIM-1 transcript can be detected in healthy subjects. The mRNA levels in urine samples from overweight subjects was slightly but not significantly higher than individuals with normal weight (Fig. 5C). NGAL is detected in injured renal tubular cells in AKI before a decrease in the glomerular filtration rate (GFR) is detectable^44,45^. The expression of NGAL was low in healthy subjects with normal weight, which is evident as judged by a representative image in Fig. 3C. No differences were found between the study groups (Fig. 5D).

The population analyzed showed positive correlations between BMI versus SBP and BMI versus DBP (Fig. 2A,B). We also found a positive but not significant correlation between BFM versus FBG and urinary protein versus FBG (Fig. 2C,D). However, individuals in the overweight group showed significantly higher systolic and diastolic blood pressure (Table 1) compared to the population with normal weight. Percentage of visceral fat was also significantly greater, which might contribute to a possible activation of systemic RAS. Although we did not find significant correlations between percentage of BFM and SBP, we did find a significant correlation between DBP and percentage of BFM (Fig. 2D), which indicates that elevated BFM might anticipate high SBP. We did not find a significant difference in FBG between the groups. Interestingly, in this study none of the subjects were hypertensive, indicating that BMI could be a predictor of incipient high blood pressure in young individuals.

Urine samples from healthy and injured kidneys contain tubular-derived cells expressing a variety of molecules that reflect the ongoing intra-renal and intra-tubular status. Urine samples represent a useful tool to detect biomarkers for kidney disease. In this study we have successfully used RNA detection in non-invasively collected urine samples to perform quantitation of mRNA transcripts for AGT, renin, IL-18, CTGF, KIM-1 and NGAL by qPCR. The qPCR technique has been widely used and validated in absolute and relative quantification of mRNA from tissues and body fluids. Transcript mRNAs levels were normalized in relation to 18S RNA using the 2^−ΔΔCт^ method. Although some reports suggest that GAPDH are preferable to 18S rRNA for urine analysis^46^, quantification and amplification of 18S rRNA ensure PCR quality and reproducibility (see “Method” section). The 18S rRNA has been used for normalization of the expression of a specific gene in urinary cell mRNA profiles for the prediction of human kidney allograft status^47^ and in inflammatory cells for the detection of variations in cytokine and inflammatory expression^48^. In our study, the 18S rRNA transcript levels were not significantly different between the patient groups and showed low variability among samples examined by melting curves in different urine samples (data not shown). Urine samples from healthy subjects were additionally used to perform non-quantitative PCR using the qPCR primer sets. Figure 3C illustrates the PCR products of each gene analyzed. We demonstrated the presence of AQP-2, which indicates that some of the desquamate epithelial cells arise from distal nephron segments. Given the criteria used for participant exclusion, it is unlikely that transcripts detected in urine reflect another source such as blood cells or small vesicles able to traverse a healthy glomerular filtration barrier.

Our findings are supported by previous studies demonstrating that injury and inflammatory markers are augmented in obesity and associated with increased oxidative stress^49^. Singh et al. showed that urinary TNF-alpha is significantly elevated in obese adolescents and correlates with urinary endothelin-1, a biomarker for endothelial dysfunction. The authors also propose that TNF-alpha may be used as a non-invasive tool to monitor the level of inflammation in obesity, which is a chronic inflammatory state^50^.

It has been suggested that changes in urinary renin levels in patients with diabetes occur independently of changes in plasma renin, indicating involvement of the activated renal RAS. Therefore, urinary renin may be a better indicator of renal RAS activity than urinary angiotensinogen or aldosterone^51^. Tang et al.^52^ demonstrated that renin levels are greater in the urine of both patients with diabetes and mice with induced diabetes type I. To examine the role of filtration and tubular reabsorption on urinary renin, the authors used mice infused with recombinant renin and lysine, which is an inhibitor of proximal tubular protein reabsorption. Lysine infusion markedly increased urinary renin levels between both nondiabetic and diabetic mice, such that there was little difference between the two groups. The authors also showed no differences between the levels of renin transcripts in micro-dissected collecting ducts of diabetic mice and that of wild-type mice, suggesting that most of the renin detected in the urine under diabetic conditions is a result of altered renin filtration and impaired reabsorption at the proximal tubules^52^.

Despite this evidence other studies have suggested that renin transcript is augmented in CD in diabetic mice^53,54^. Then, early detection of kidney injury markers and other RAS components in intra-tubular epithelial cells along the nephron may help to establish an imminent risk of renal disease in overweight young adults. Moreover, BMI is easy to measure in pediatric and young populations, and the non-invasive collection of urine can provide information on renal status using qPCR. This may represent a new strategy for CKD risk screening in the adult. As suggested by other studies, the use of BMI is also recommended to predict and screen hypertension^55^.

Despite the limitation of small sample size in our study, we observed a clear phenomenon that has been described in several recent clinical studies that employed large numbers of patients and in which the same association between BMI and BP was observed. Our study adds further molecular information to explore the intra-renal status of the RAS and tissue injury. This additional analysis might be adopted as a complementary approach in future studies using large sample numbers.

In conclusion, we have demonstrated that SBP and DBP augmented significantly and linearly across BMI levels in a normotensive population of young adults. Using a non-invasive methodology, we found that overweight individuals have higher mRNA levels of urinary renin and AGT, a reflection of intra-tubular RAS activation. This group also showed high expression of IL-18 and CTGF. This suggests that BMI may have direct effects on blood pressure and the activation of intra-tubular RAS, which can lead to kidney injury. Therefore, BMI likely poses a risk factor for incipient kidney disease even in normotensive young adults.

## Supplementary information

Supplementary Information.
