# Supplementary material for: Augmented transcripts of kidney injury markers and renin angiotensin system in urine samples of overweight young adults
Source: Sci Rep. 2020 Dec 3;10:21154. doi: 10.1038/s41598-020-78382-3 (PMC7713175; doi:10.1038/s41598-020-78382-3)
Supplement: Supplementary file 1 — Supplementary Information. [file 41598_2020_78382_MOESM1_ESM.pptx]

## Slide 1
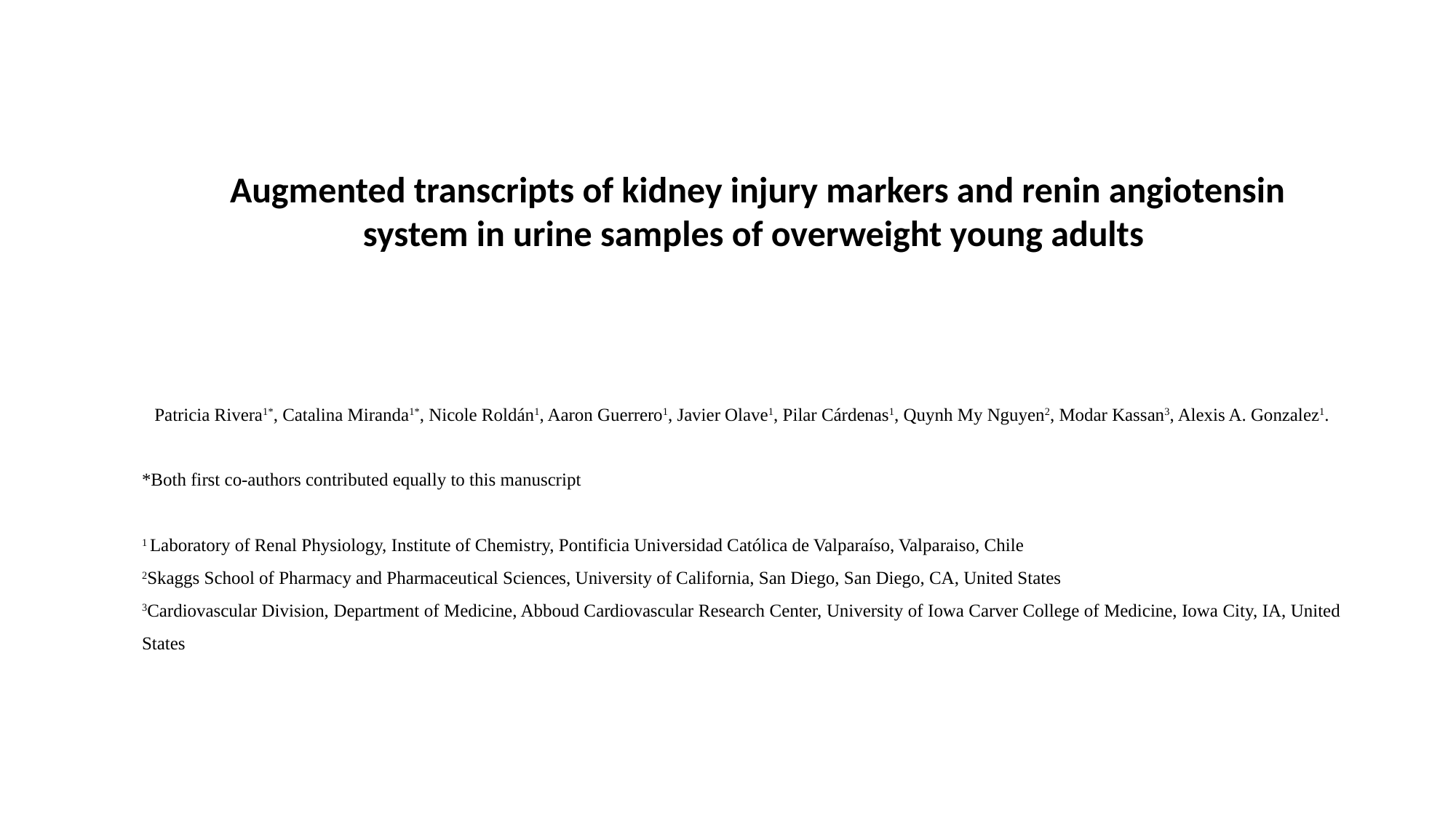

Augmented transcripts of kidney injury markers and renin angiotensin system in urine samples of overweight young adults
Patricia Rivera1*, Catalina Miranda1*, Nicole Roldán1, Aaron Guerrero1, Javier Olave1, Pilar Cárdenas1, Quynh My Nguyen2, Modar Kassan3, Alexis A. Gonzalez1.
*Both first co-authors contributed equally to this manuscript
1 Laboratory of Renal Physiology, Institute of Chemistry, Pontificia Universidad Católica de Valparaíso, Valparaiso, Chile
2Skaggs School of Pharmacy and Pharmaceutical Sciences, University of California, San Diego, San Diego, CA, United States
3Cardiovascular Division, Department of Medicine, Abboud Cardiovascular Research Center, University of Iowa Carver College of Medicine, Iowa City, IA, United States

## Slide 2
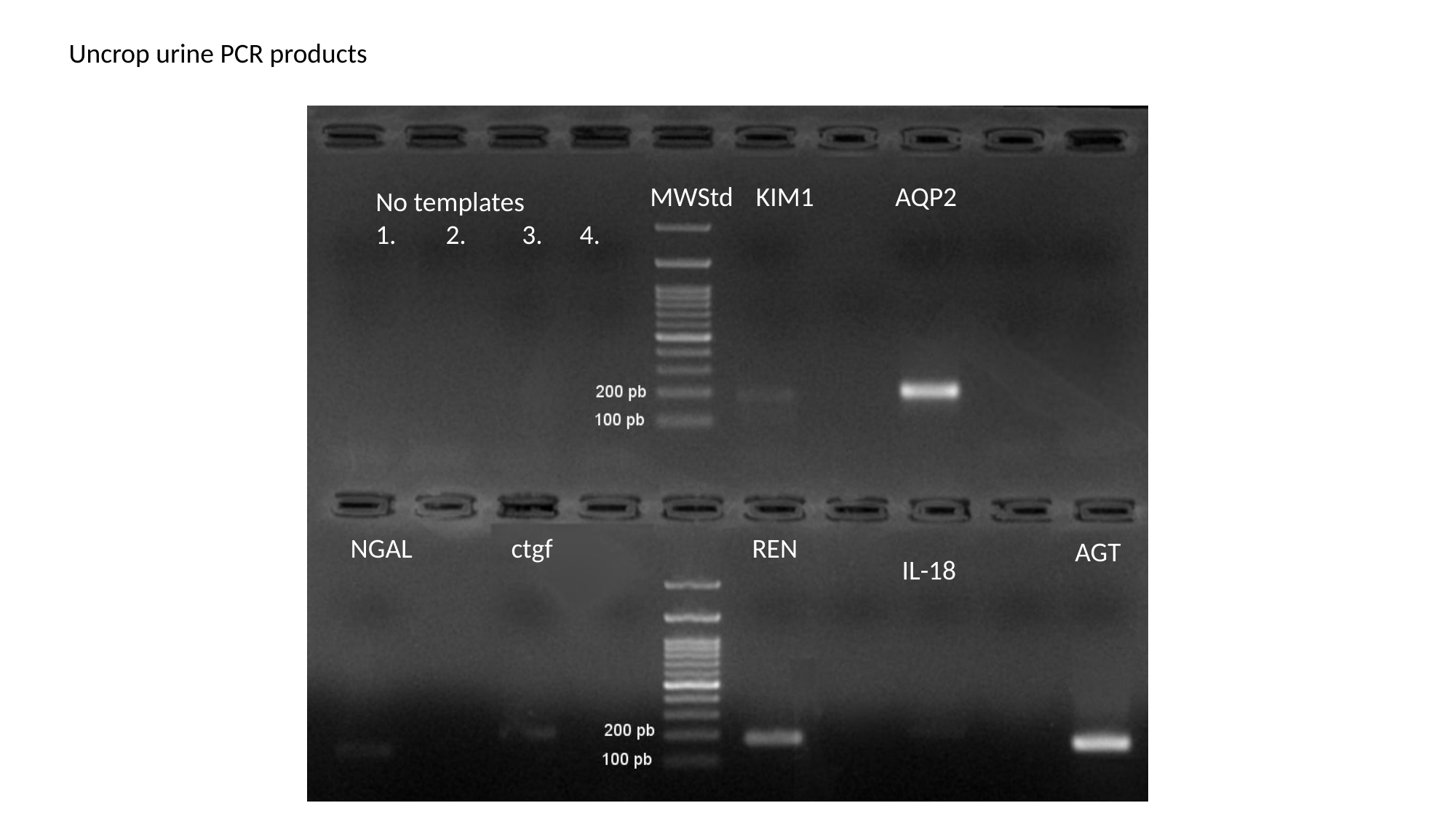

Uncrop urine PCR products
MWStd
AQP2
KIM1
No templates
1. 2. 3. 4.
NGAL
ctgf
REN
AGT
IL-18
